# Polymorphism of di­methyl­amino­borane N(CH_3_)_2_-BH_2_

**DOI:** 10.1107/S2052520621001979

**Published:** 2021-03-26

**Authors:** Alexander Bodach, Thomas Bernert, Michael Fischer, Morten Brix Ley, Claudia Weidenthaler

**Affiliations:** a Heterogeneous Catalysis, Max-Planck-Institut für Kohlenforschung, Kaiser-Wilhelm-Platz 1, Mülheim an der Ruhr, 45470, Germany; b Crystallography/Geosciences, University Bremen, Klagenfurter Str., Bremen, 28359, Germany; c MAPEX Center for Materials and Processes, University of Bremen, Bremen, 28359, Germany

**Keywords:** *in situ*, powder diffraction, di­methyl­amnoborane, hydrogen storage, polymorphism

## Abstract

De­hydro­coupling of the adduct of di­methyl­amine and borane, NH(CH_3_)_2_-BH_3_ leads to di­methyl­amino­borane with formal composition N(CH_3_)_2_-BH_2_. The structure of this product depends on the conditions of the synthesis; it may crystallize either as a dimer in a triclinic space group forming a four-membered ring [N(CH_3_)_2_-BH_2_]_2_ or as a trimer forming a six-membered ring [N(CH_3_)_2_-BH_2_]_3_ in an orthorhombic space group. Due to the denser packing, the six-membered ring in the trimer structure should be energetically more stable than the four-membered ring. The triclinic structure is stable at low temperatures. Heating the triclinic phase above 290 K leads to a second-order phase transition to a new monoclinic polymorph. While the crystal structures of the triclinic and orthorhombic phases were already known in the literature, the monoclinic crystal structure was determined from powder diffraction data in this study. Monoclinic di­methyl­amino­borane crystallizes in space group *C*2/*m* with the boron and nitro­gen atoms located on the mirror plane, Wyckoff position 4*i*, while the carbon and hydrogen atoms are on the general position 8*j*.

## Abstract 2

The intermediate N(CH_3_)_2_-BH_2_ aggregates either to dimers or trimers. Herein, the stability of the adducts is investigated experimentally and computationally, and the monoclinic crystal structure of [N(CH_3_)_2_-BH_2_]_2_ is provided.

## 1. Introduction   

As fossil fuels diminish, new energy carriers have to be developed to maintain our living standards, especially our mobility. Hydrogen is an energy carrier that can be used in combination with fuel cell technology. However, several challenges remain to be resolved in the storage and generation of hydrogen before hydrogen becomes an efficient, cheap, safe and clean energy carrier (Eberle *et al.*, 2009 ▸; Weidenthaler & Felderhoff, 2011 ▸; Ley *et al.*, 2014 ▸). The storage of hydrogen is possible using several techniques: high-pressure storage in gas tanks, liquefaction, physisorption in high surface area materials such as metal–organic frameworks (MOFs) or chemisorption in metal hydrides or complex metal hydrides. High-pressure storage is currently the state-of-the-art method for automobile applications, *e.g.* in the Toyota Mirai [*m*(H_2_) = 5 kg H_2_, *p*(H_2_) = 700 bar] (Yoshida & Kojima, 2015 ▸). Mercedes-Benz has launched a fuel cell car (GLC F-CELL) with pressurized hydrogen combined with a battery. However, the design of the vehicle is limited by the tank design and utilized materials (Weidenthaler & Felderhoff, 2011 ▸; Ley *et al.*, 2015 ▸). High-pressure technology defines future infrastructure requirements and any other hydrogen storage system should preferably be compatible with this technology (Weidenthaler & Felderhoff, 2011 ▸). Different metal hydrides are considered for chemical storage of hydrogen: metal hydrides, complex hydrides (*e.g.* borohydrides and aluminium hydrides), metal imides/amides and amine borane adducts/amino­boranes or amino­alanes (Weidenthaler & Felderhoff, 2011 ▸; Ley *et al.*, 2015 ▸, 2016 ▸; Chen *et al.*, 2002 ▸; Bernert *et al.*, 2016 ▸). Metal hydrides often have low gravimetric hydrogen capacities, whereas complex metal hydrides may suffer from poor reversibility (Ley *et al.*, 2014 ▸). Unfortunately, metal amides need high reaction temperatures and may release small amounts of ammonia, reducing the capacity and poisoning the fuel cells (Jepsen *et al.*, 2014 ▸). De­hydrogenation reactions based on H^δ+^ and H^δ−^ interactions are a promising strategy for reversible hydrogen release, storage and activation, *e.g.* in ammonia borane (Staubitz *et al.*, 2010 ▸), amine–metal borohydrides (Jepsen, Ley, Filinchuk * et al.*, 2015 ▸; Jepsen, Ley, Cerny *et al.*, 2015 ▸; Castilla-Martinez *et al.*, 2020 ▸), and solutions containing frustrated Lewis pairs (FLPs) (Welch *et al.*, 2006 ▸; Stephan & Erker, 2010 ▸) and even solid FLPs (Bowden *et al.*, 2020 ▸). Notably, amino­boranes offer high gravimetric hydrogen content, which may be released stepwise. Amine boranes as monomeric precursors are mainly synthesized either from diborane, B_2_H_6_, or borane tetra­hydro­furan complex, BH_3_·thf, and an amine (Staubitz *et al.*, 2010 ▸) or from a borohydride and ammonium chloride, which release one equivalent of hydrogen during salt metathesis (Jaska *et al.*, 2003 ▸).

Amino­boranes can aggregate to form four- or six-membered rings *via* de­hydro­coupling of amine borane adducts. In the case of six-membered rings, triboratrizanes can finally be de­hydrogenated to triboratriazines (aromatic six-membered rings) (Jaska *et al.*, 2003 ▸, 2001 ▸). Based on recent investigations on alkyl­amino­alanes, also forming four- or six-membered rings (Bernert *et al.*, 2016 ▸; Ley *et al.*, 2016 ▸; Downs *et al.*, 1992 ▸), alkyl­amino­boranes are structurally very interesting. Depending on the reaction conditions, the de­hydro­coupling of di­methyl­amine borane, NH(CH_3_)_2_-BH_3_, either leads to the dimeric form [N(CH_3_)_2_-BH_2_]_2_ (Jaska *et al.*, 2001 ▸) (CCDC refcode DMABDI01) consisting of a four-membered ring or the trimeric form [N(CH_3_)_2_-BH_2_]_3_ (Trefonas *et al.*, 1961 ▸) consisting of a six-membered ring (see Scheme 1, which also shows the de­hydro­coupling of di­methyl­amine borane adduct to di­methyl­amino­borane followed by dimerization and trimerization). [N(CH_3_)_2_-BH_2_]_3_ (CCDC refcode DMABTR) has so far only been produced by the addition of *nido*-pentaborane, B_5_H_9_ (Burg, 1957 ▸; Campbell & Johnson, 1959 ▸; Burg & Sandhu, 1967 ▸). During catalytic de­hydro­coupling reactions, [N(CH_3_)_2_−BH_2_]_2_ is often the main product, while [N(CH_3_)_2_-BH_2_]_3_ may also form in low quantities (Jaska *et al.*, 2003 ▸, 2001 ▸; Rossin & Peruzzini, 2016 ▸). The mechanism remains unknown for the formation of either dimers or trimers. Di­methyl­amino­borane is a compound of a group-13 element hydride and an amine. It can crystallize as a dimer or a trimer, with both crystal structures already determined, see above. To get some insight and to identify parameters that influence the formation of the dimeric and trimeric di­methyl­amino­borane, we investigated the phase stability of dimeric di­methyl­amino­borane depending on the temperature and the energy of both forms of di­methyl­amino­borane by experimental and computational methods
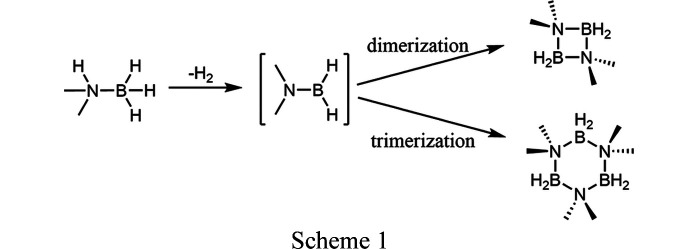
.

## 2. Experimental   

### 2.1. Preparation   

All preparations and manipulations of the compounds were performed under dry argon atmosphere using Schlenk techniques or a glovebox in either flame dried glassware or autoclaves. Di­ethyl­ether (Et_2_O), tetra­hydro­furan (thf) and diglyme (Sigma-Aldrich, 98%) were dried using sodium and benzo­phenone and distilled prior to use (< 10 p.p.m. H_2_O). Sodium borohydride NaBH_4_ (Sigma-Aldrich, 99%) and di­methyl­amine hydro­chloride NH_2_(CH_3_)_2_Cl (Sigma-Aldrich, 99%) were used as received. In contrast to the transition metal catalysed de­hydro­coupling synthesis (Jaska *et al.*, 2003 ▸), the synthesis was performed at higher temperatures without a catalyst. Thermolytic de­hydro­coupling experiments were conducted in a 36 ml steel autoclave with polytetra­fluoro­ethyl­ene (PTFE) inlet, equipped with a pressure sensor and a thermocouple. It is worth noting that the high sublimation rate and a low melting point of [(CH_3_)_2_N-BH_2_]_2_ make the handling of this compound challenging. Thermolytic de­hydro­coupling in autoclaves often led to a significant amount of sample being trapped between the autoclave wall and inlet, thereby making a precise determination of the yield difficult. NaBH_4_ (2.656 g, 70.2 mmol) and NH_2_(CH_3_)_2_Cl (5.705 g, 70.0 mmol) were mixed in diglyme (100 ml) and refluxed overnight at 443 K. During this time, large (needle-like) crystals grew in the bulb condenser.

Small impurities from diglyme (< 1%) were observed in the ^1^H NMR spectrum and the IR data (marked with an asterisk), while all analytical data are consistent with those of Jaska *et al.* (2001 ▸, 2003 ▸). ^1^H (300 MHz, CDCl_3_) δ (p.p.m.) = 1.96–3.20 (*q*, br, BH_2_), 2.39 (*s*, CH_3_) ^11^B (96 MHz, CDCl_3_) δ (p.p.m.) = 4.75 (*t*, *J* = 110 Hz) ^13^C (75 MHz, CDCl_3_) δ (p.p.m.) = 51.77. The melting point obtained by DSC is *T*_m_ = 348 K.

IR (cm^−1^): 794 (*m*), 833 (*m*), 877 (*w*), 919 (*m*), 1004 (*s*), 1054 (*w*), 1099 (*w*), 1139 (*s*), 1166 (*m*), 1187* (*s*), 1213 (*m*), 1313 (*w*), 1382* (*w*), 1450 (*w*), 1469 (*w*), 2356 (*m*), 2418 (*m*), 2881 (*w*), 2939 (*w*), 2975* (*w*).

### 2.2. NMR and infrared spectroscopy   

Nuclear magnetic resonance (NMR) spectra (^1^H, ^11^B, ^13^C) were recorded on a BRUKER AVIII 300 nanobay spectrometer at 298 K in CDCl_3_. All chemical shifts, δ, are given in p.p.m., referenced to the residual peak of the deuterated solvent according to Fulmer *et al.* (2010 ▸).

Infrared spectra were recorded on a NICOLET MAGNA IR 560 ESP system. Measurements were performed with an ATR unit.

### 2.3. Thermal analysis   

The melting points of the synthesized samples were determined using a SETARAM Micro DSC VII calorimeter with closed steel crucibles, filled under argon atmosphere. During the measurement, the sample (∼20 mg) was heated at a rate of 1 K min^−1^ from ambient temperature to 373 K. The sublimation point of [(CH_3_)_2_N-BH_2_]_3_ was determined using a METTLER TOLEDO TGA/DSC 1 STAR system. The sample was packed in an aluminium crucible and heated from ambient temperature to 573 K at a heating rate of 5 K min^−1^.

### 2.4. X-ray powder diffraction   

Di­methyl­amino­borane was sealed in a borosilicate glass capillary with an inner diameter of 0.5 mm. X-ray powder diffractograms were recorded using Mo *K*α_1_ radiation (λ = 0.7093 Å) on a Stoe Stadi-P powder diffractometer in Debye–Scherrer geometry. Temperature-dependent X-ray powder diffraction was performed in a temperature range between 173 and 318 K. Cooling was achieved with an Oxford Cryostream 700 using liquid nitro­gen with a cooling rate of 6 K min^−1^. The measurements were performed at ambient pressure using a curved Ge (111) monochromator in the primary position and a strip detector (MYTHEN 1K). For the temperature-dependent Rietveld refinements using the program *GSAS* (Larson & Von Dreele, 2004 ▸), a pseudo-Voigt function was employed (Thompson *et al.* (1987 ▸) in conjunction with an asymmetry correction (Finger *et al.*, 1994 ▸). A total of 3993 data points were used to refine 52 parameters. In the case of the structure refinement with a triclinic cell, including the refinement of the fractional coordinates of the carbon, boron and nitro­gen atoms, 46 restraints were applied. All C—N bond lengths were set to 1.48 Å and the N—B bond lengths to 1.6 Å. Furthermore, all bond angles were restrained to physically meaningful values. The hydrogen atoms were ignored during these Rietveld refinements. In order to correct preferred orientation effects resulting from the crystallization in a capillary, intensity correction according to March–Dollase (Dollase, 1986 ▸; March, 1932 ▸) was applied. The GSAS weighting factor for the restraints was gradually decreased during the refinement. The temperature-dependent measurements were refined using shell scripts based on gsaslanguage (Vogel, 2011 ▸). For refinement of the monoclinic *C*-centred cell, the Rietveld refinement based on the determined structure is used as a starting model for the temperature-dependent refinements.

The monoclinic crystal structure (including hydrogen atoms) was solved using simulated annealing with *DASH* (David *et al.*, 2006 ▸) based on the molecular structure described by Jaska *et al.* (2001 ▸) and refined with the program package *TOPAS6.0* (Bruker, 2017 ▸) and physically meaningful restraints and one atomic displacement parameter (ADP) for all non-hydrogen atoms, while the ADP of the hydrogen atoms was set to be 1.2 times the non-hydrogen atom ADP. The final parameters are given in Table 1 ▸.

### 2.5. Computational details   

Plane-wave DFT calculations were performed using the *CASTEP* software, version 7 (Clark *et al.*, 2005 ▸), employing norm-conserving on-the-fly pseudopotentials. Within this study, the Perdew–Burke–Ernzerhoff (PBE) exchange correlation functional (Perdew *et al.*, 1996 ▸) was augmented using a pairwise dispersion correction, employing both the D2 correction developed by Grimme (2006 ▸) and the TS correction by Tkatchenko & Scheffler (Tkatchenko & Scheffler, 2009 ▸). Full geometry optimization was performed on the crystal structures of dimeric di­methyl­amino­borane from Jaska *et al.* (2001 ▸) and trimeric di­methyl­amino­borane (Trefonas *et al.*, 1961 ▸) employing a plane wave cut-off energy of 800 eV. All optimizations used the following convergence thresholds: change in total energy below 10^−6^ eV, largest residual force below 0.005 eV Å^−1^, largest displacement below 5 × 10^−4^ Å, largest component of the stress tensor smaller than 0.001 GPa. For the triclinic and monoclinic structures of dimeric di­methyl­amino­borane, a 4 × 4 × 3 grid of *k*-points was used, whereas a 2 × 2 × 2 grid was used for the trimeric ortho­rhombic form. Phonon calculations for monoclinic di­methyl­amino­borane were performed using a 4 × 4 × 3 grid of *q*-points. These calculations were carried out in the framework of variational density functional perturbation theory (DFPT) (Refson *et al.*, 2006 ▸). For the DFPT calculations, the structure, including unit-cell parameters, was first optimized with the PBE-TS functional. Then the atomic coordinates were relaxed using the PBE functional, as the version of CASTEP used supports DFPT calculations only for functionals without dispersion correction.

## 3. Results and discussion   

### 3.1. Phase transition of dimeric di­methyl­amino­borane from triclinic to monoclinic   

**Figure 1:**
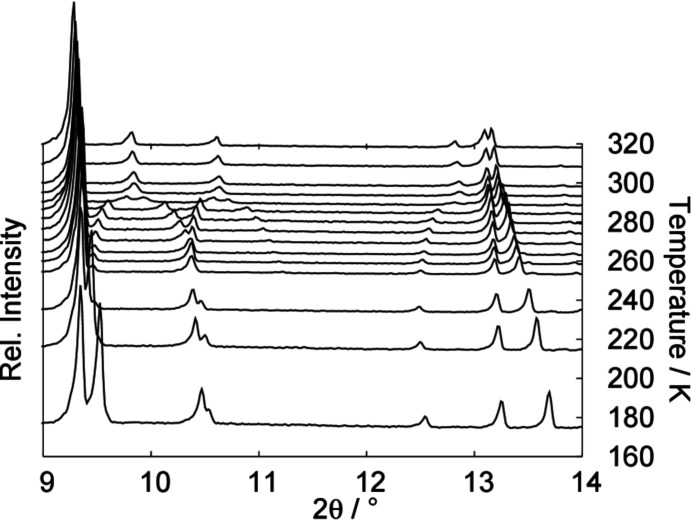
Selection of X-ray powder diffraction patterns of di­methyl­amino­borane collected during heating from 173 K to 318 K. The phase transformation from the low-temperature triclinic structure to the monoclinic structure occurs above 270 K.

At 100 K, dimeric di­methyl­amino­borane crystallizes in the triclinic space group 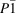 with *a* = 5.8330 (7) Å, *b* = 6.029 (10) Å, *c* = 6.2400 (10) Å, α = 80.372 (8)°, β = 81.533 (10)° and γ = 65.942 (8)° and *V* = 196.80 (5) Å^3^ (Jaska *et al.*, 2001 ▸). At elevated temperature, di­methyl­amino­borane undergoes a phase transition from the triclinic to a monoclinic structure. Fig. 1 ▸ shows the *in situ* X-ray powder diffraction patterns of di­methyl­amino­borane collected in the temperature range between 173 K and 318 K.

The crystal structure of the monoclinic phase could not be determined from single-crystal data due to the high sublimation rate of di­methyl­amino­borane. Therefore, the crystal structure was solved from X-ray powder diffraction data and refined with Rietveld methods. The crystal structure of monoclinic di­methyl­amino­borane is described in space group *C*2/*m* with *a* = 6.2314 (3) Å, *b* = 11.0574 (7) Å, *c* = 6.2759 (4) Å and β = 98.754 (3)°. The space group and unit-cell parameters of monoclinic di­methyl­amino­borane were already determined by Schapiro (1962 ▸; CCDC refcode DMABDI) to be *a* = 6.24 Å, *b* = 11.07 Å, *c* = 6.28 Å, β = 98.8°, and *V* = 428.7 Å^3^ at ambient temperature, but no fractional coordinates were provided. The results of the structure determination from X-ray powder data of this study are in good agreement with the unit-cell parameters from Schapiro (1962 ▸) (Table 1 ▸).

**Figure 2:**
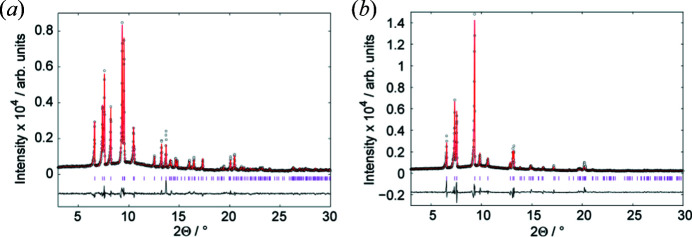
Rietveld refinements of the powder diffraction data of di­methyl­amino­borane (*a*) in the triclinic structure measured at 173 K and (*b*) the monoclinic structure measured at 293 K. Black dots: measured intensities *I*_o_, red line: calculated intensities *I*_c_, black line: difference pattern *I*_o_ − *I*_c_. The tick marks correspond to the positions of the Bragg reflections.

Rietveld refinements of the powder diffraction data of the triclinic [Fig. 2 ▸(*a*)] and the monoclinic [Fig. 2 ▸(*b*)] phases confirm that di­methyl­amino­borane is the only crystalline compound in the sample and that the crystal structure determination from X-ray powder diffraction data on the monoclinic sample is representative for the bulk material.

The molecular structure of di­methyl­amino­borane is similar in the triclinic and the monoclinic phases. Without considering the hydrogen atoms, the symmetry of the di­methyl­amino­borane molecule is approximately *D*_2*h*_. In the triclinic structure with space group 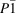, the centre of inversion is directly located within the B_2_N_2_ four-membered ring. All atoms are located on the general position 2*i*, leading to point group *C_i_* for the molecule. However, in the monoclinic structure with space group *C*2/*m*, the boron and nitro­gen atoms are located on a mirror plane, Wyckoff position 4*i*, while the carbon and hydrogen atoms are positioned at general position 8*j*, which leads to the point group for the molecule of *C*_2*h*_.

**Figure 3:**
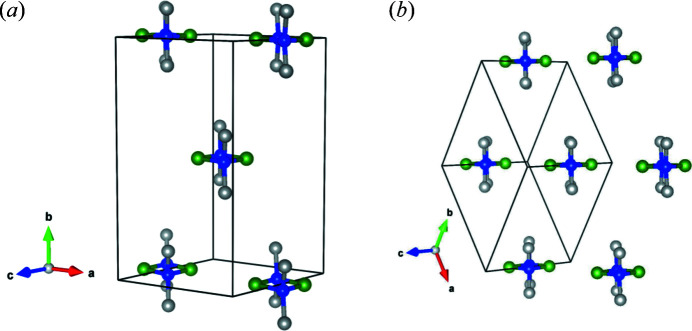
Crystal structure of dimeric di­methyl­amino­borane: (*a*) monoclinic *C*2/*m*, along [101] and (*b*) triclinic 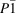, along [111] (grey: carbon atoms, green: boron atoms and blue: nitro­gen atoms). Hydrogen atoms are omitted for clarity.

The arrangement of the molecules in the triclinic and the monoclinic phases is a distorted hexagonal close-packed (Fig. 3 ▸). The hexagonal layers are in the 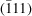 plane in the monoclinic structure, while the stacking direction is 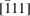. The layers in the triclinic structure as described by Jaska *et al.* (2001 ▸) are in the (111) plane with stacking along [111]. The triclinic structure can be considered a *translationengleiche* subgroup of index 2 (*t*2) of the monoclinic high-temperature phase. The similarity between both crystal structures becomes apparent if the monoclinic *C*-centred cell is transformed into the corresponding primitive cell. In the standard setting with *a* = *b*, *c*, α = β and γ (Hahn *et al.*, 2005 ▸), the primitive cell is given by: (***a***_p_,***b***_p_,***c***_p_) with *a* = *b* = 6.36 Å α = β = 94.32°, γ = 121.10°.

The coordinates of the *C*-centred cell were then transformed with the inverse matrix of ***P*** and an origin shift of (½, 0, ½) [equation (1)]. The unit-cell given by Jaska *et al.* (2001 ▸), with ***a***_J_, ***b***_J_, ***c***_J_, can then be transformed into a cell similar to the primitive unit-cell (***a***_p_,***b***_p_,***c***_p_) by equation (1)
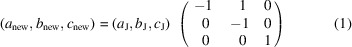
with *a* = 5.8330 Å, *b* = 6.4576 Å, *c* = 6.2400 Å, α = 91.330°, β = 98.470° and γ = 121.510°. Rietveld refinements were performed with the powder diffraction data measured between 173 and 318 K, using the transformed cell from Jaska *et al.* (2001 ▸) by equation (1).

**Figure 4:**
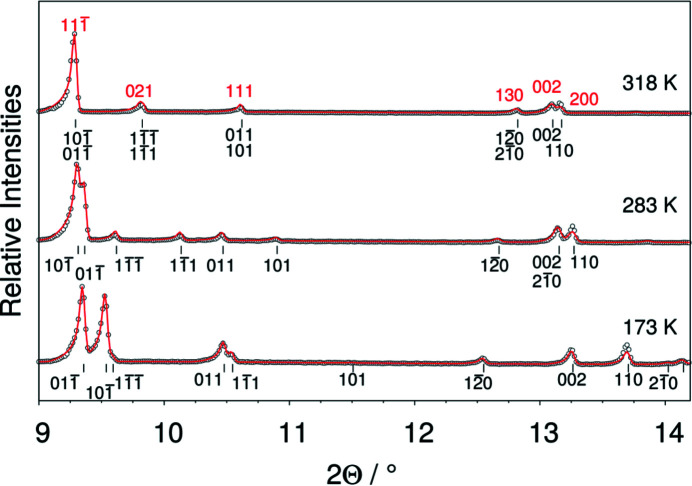
Diffraction patterns of di­methyl­amino­borane measured at 173 K, 283 K, and 318 K. Black dots: measured intensity, red line: calculated intensity. The tick marks indicate the positions of the Bragg reflections. The black indices correspond to the triclinic cell obtained by the transformation shown in equation (1), whereas the red indices correspond to the *C*-centred monoclinic cell.

Fig. 4 ▸ shows the powder diffractograms of di­methyl­amino­borane collected at 173 K, 283 K and 318 K with the indices of the reflections [black: triclinic cell obtained by equation (1) and red: reflection indices of the monoclinic *C*-centred cell]. The dependence of the diffraction angle of the reflections as a function of the temperature reveals that pairs of reflections, *e.g.* 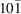 and 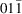, 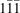 and 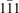, 011 and 101 and the 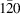 and 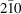 shift to the same angle, *i.e.* the same *d*-spacing, which then transform into the equivalent reflections of the monoclinic *C*-centred cell.

**Figure 5:**
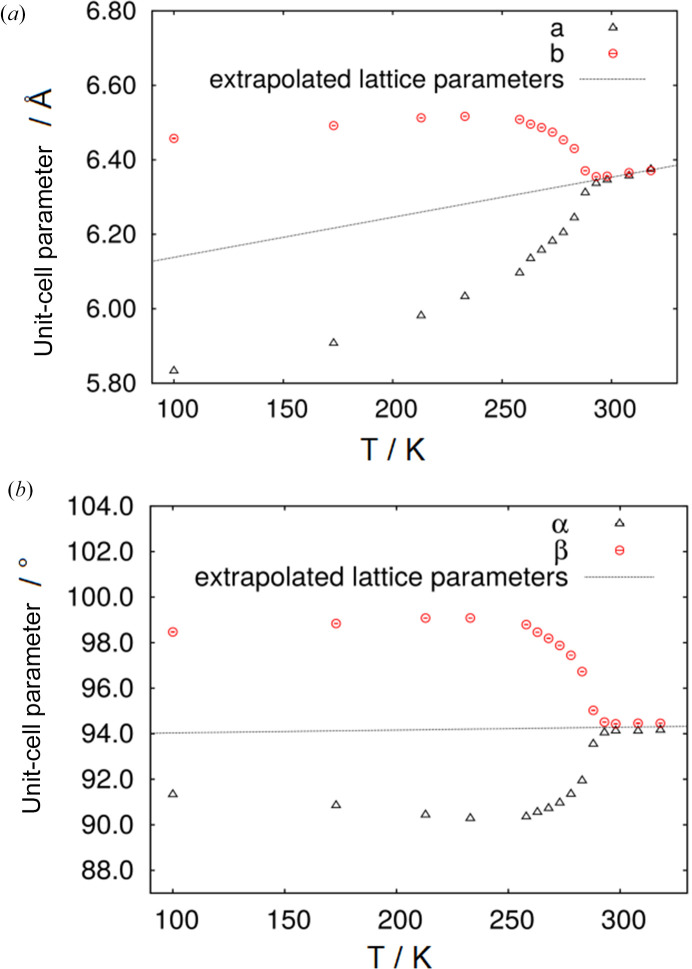
Change of the unit-cell parameters (*a*) *a* and *b* and (*b*) α and β of di­methyl­amino­borane during heating from 173 to 318 K. The unit-cell parameters at 100 K are taken from Jaska *et al.* (2001 ▸) and transformed according to equation (1) (the error bars are smaller than the symbol sizes). The dashed lines are the extrapolated unit-cell parameters of the monoclinic phase.

From Rietveld refinements of the temperature-dependent diffraction data, unit-cell parameters as a function of temperature were obtained. Fig. 5 ▸(*a*) shows the change of the unit-cell parameters *a* and *b* and angles α and β [Fig. 5 ▸(*b*)] of the triclinic cell as a function of the temperature. Between 245 and 290 K, rearrangement of the molecules takes place. At 245 K, the phase transition starts, which results at 290 K in the formation of the monoclinic phase. At 290 K, the unit-cell parameters fulfil the conditions *a* = *b* and α = β, which implies the primitive cell of the above-mentioned *C*-centred monoclinic unit cell (Hahn *et al.*, 2005 ▸). Once this relation between the unit-cell parameters is established, it does not change by a further increase of the temperature.

**Figure 6:**
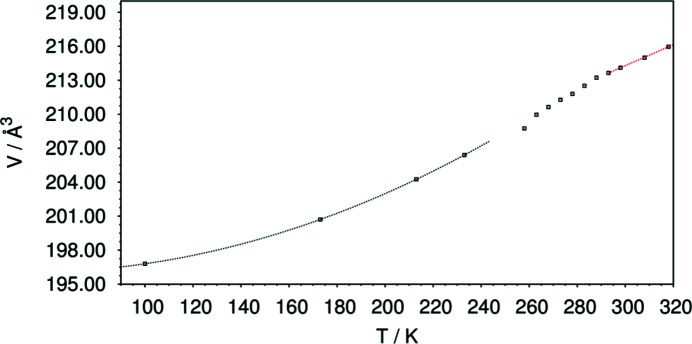
Change of the unit-cell volume of di­methyl­amino­borane as a function of the temperature. Black squares: measured data (error bars are smaller than the symbol size), dashed line (blue): fit of a second-order polynomial on the unit-cell volumes of the triclinic phase and dashed line (red): linear fit on the unit-cell volumes of the monoclinic phase.

The changes of the thermal expansion are also represented by the changes of the unit-cell volume with temperature (Fig. 6 ▸). From 100 K to 245 K, the unit-cell volume changes are based on the thermal expansion of the triclinic phase, while the change of the unit-cell parameters in the temperature range 290–318 K is caused by the thermal expansion of the monoclinic structure, Fig. 6 ▸.

**Figure 7:**
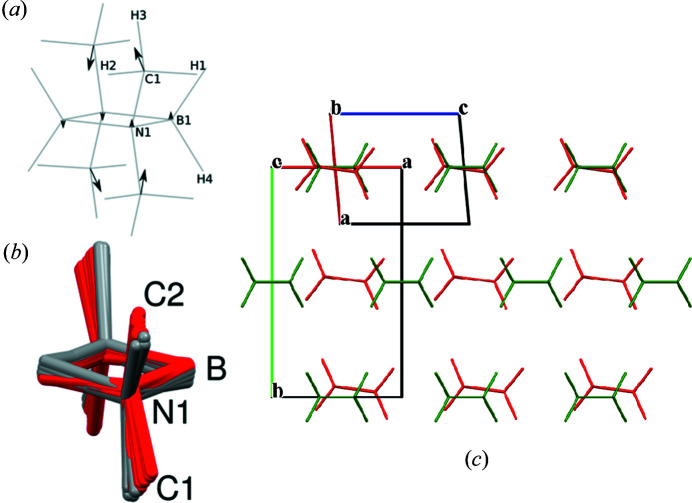
(*a*) Scheme of the low-frequency *B_g_* mode eigenvectors at 8.06 cm^−1^, obtained from phonon calculations. The length of the arrows was chosen according to the magnitude of eigenvectors from the di­methyl­amino­borane four-membered ring obtained from the phonon calculations. The numbering scheme is in accordance with the DFPT calculations. The eigenvectors of the hydrogen atoms were omitted. (*b*) Evolution of the atomic positions of di­methyl­amino­borane in the temperature range from 173 to 288 K (red) and from 293 to 318 K (grey), determined from the Rietveld refinements. Hydrogen atoms are omitted for clarity. (*c*) An overlay of packing motifs from the triclinic phase (Jaska *et al.*, 2001 ▸) in red and the new monoclinic polymorph in green. The origin was chosen to have a suitable overlay of two molecules (mid left). Hydrogen atoms are omitted for clarity.

To further investigate the temperature-dependent shift of the molecule within the unit cell, density functional perturbation theory (DFPT) calculations were performed. Fig. 7 ▸(*a*) shows the eigenvectors of a low-frequency *B_g_* mode with a frequency of 8.06 cm^−1^ at Γ and with imaginary frequencies close to the Γ point. Fig. 7 ▸(*b*) shows the overlay of the atomic positions obtained from Rietveld refinements of powder diffraction data collected from 173 K to 318 K. The red atom positions are taken from the refinement of data collected in the temperature range between 173 and 283 K. The positions displayed in red belong to the triclinic phase. In contrast, the grey positions were obtained from the refinement of data collected in the temperature range where the monoclinic phase is stable. Fig. 7 ▸ indicates a significant contribution of this low-frequency *B_g_* mode to the temperature-dependent shift of the molecule, as expected from the Bose–Einstein statistics, predicting a high population of low-frequency modes. From Fig. 7 ▸(*b*), it can be concluded that cooling of the monoclinic phase leads to the "freezing of molecular vibrations", reducing the symmetry from 2/*m* to 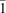 in the triclinic structure. This behaviour fits the *B_g_* irreducible representation in point group *C*_2*h*_. Hence in an elastic transition from point group *C*_2*h*_ to *C_i_*, the strain is represented by *B_g_* (Aubry & Pick, 1971 ▸). Fig. 7 ▸(*c*) visualizes the slightly shifted molecules in a packing diagram of the triclinic (red) and monoclinic (green) polymorph based on origins chosen to overlay two molecules. Compared to the monoclinic structure, the molecules in the triclinic structure are tilted, which is also indicated by the eigenvector calculations. In addition, the molecules in the triclinic packing are shifted with respect to the monoclinic arrangement. These differences can be explained by stress during the phase transformation indicated by the eigenvector of a low-frequency *B_g_* mode with a frequency of 8.06 cm^−1^ at Γ [Fig. 7 ▸(*a*)] from the phonon calculations.

Therefore, the *B_g_* mode at 8.06 cm^−1^ is a suitable candidate for a soft-optical mode, coupled to the components of the spontaneous strain and driving this phase transition (Unruh, 1995 ▸). This interpretation is corroborated by the generation of a distorted structure according to the eigenvectors of this mode: after DFT optimization (PBE-TS functional), this structure is essentially indistinguishable from the optimized structure that started from the experimental triclinic structure (Jaska *et al.*, 2001 ▸). A comparison of both DFT-optimized triclinic structures with the *COMPSTRU* program (de la Flor *et al.*, 2016 ▸) delivered a measure of similarity as defined by Bergerhoff *et al.* (1999 ▸) of 0.003 (0 = identical structures). This result shows that the triclinic phase can indeed be reached from the monoclinic structure through displacements associated with the *B_g_* mode at 8.06 cm^−1^. The DFT energy difference between the triclinic and monoclinic structure amounts to −1.67 kJ mol^−1^ per formula unit (p.f.u.) in calculations with the PBE-D2 functional, and to −1.95 kJ mol^−1^ (p.f.u.) when using the PBE-TS functional. While these values appear to be plausible for a phase transition occurring near room temperature, a DFT-based prediction of the transition temperature would require a calculation of the vibrational contributions to the free energy for both phases, which is precluded by the presence of a mode with imaginary frequency in the monoclinic phase.

### 3.2. DFT calculation and the stability of the dimer and the trimer   

**Figure 8:**
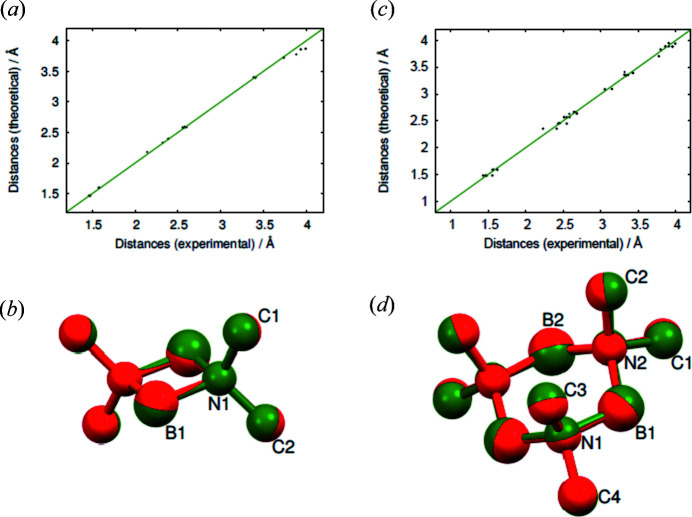
(*a*) Comparison of the experimental interatomic distances of the dimeric di­methyl­amino­borane from Jaska *et al.* (2001 ▸) to the results of the DFT calculation of this study. (*b*) Overlay of the experimental molecular structure (red) of the dimeric di­ethyl­amino­borane with the calculated molecular structure (green). (*c*) Comparison of the experimental interatomic distances of the trimeric di­methyl­amino­borane from Trefonas *et al.* (1961 ▸) to the results of the DFT calculation of this study. (*d*) Overlay of the experimental molecular structure of the trimeric di­ethyl­amino­borane (red) with the calculated molecular structure (green).

Table 2 ▸ compares the experimental unit-cell parameters with the results obtained from geometry optimization using the dispersion-corrected DFT calculations. The DFT calculations were performed in the athermal limit without considering vibrations of the atoms. Therefore, the unit-cell parameters of the triclinic phase were extrapolated to 0 K in order to approximate the experimental values to the conditions of the calculations as described, for example, by Liu *et al.* (2013 ▸) or Schimka *et al.* (2013 ▸). Use of the PBE-TS functional leads to good agreement with experimental unit-cell parameters for the triclinic structure, whereas the PBE-D2 functional underestimates the unit-cell dimensions rather significantly. The deviations obtained for the trimeric orthorhombic phase are higher than those for the dimeric triclinic phase. This could be a temperature effect. Fig. 8 ▸(*a*) shows the comparison of the theoretical and experimental interatomic distances in the triclinic structure of dimeric di­methyl­amino­borane. The distances for the calculated and experimental structures do not consider the hydrogen atoms. For both the dimeric [Figs. 8 ▸(*a*) and 8 ▸(*b*)] and the trimeric structures [Figs. 8 ▸(*c*) and 8 ▸(*d*)], just the results of the PBE-TS calculations are shown. For the experimental structure of the dimeric di­methyl­amino­borane, the extrapolated unit-cell parameters were used. From the comparison of the calculated and experimental distances for the trimeric di­methyl­amino­borane [Fig. 8 ▸(*c*)], it can be seen that the scatter of the distances is higher compared to those of the dimeric di­methyl­amino­borane [Fig. 8 ▸(*a*)]. However, the comparison of the molecular structures of the trimer [Fig. 8 ▸(*d*)] and also of the dimer [Fig. 8 ▸(*b*)] shows an excellent agreement.

Interestingly, di­methyl­amino­borane can exist as a trimer, which forms a six-membered ring and a dimer, which forms a four-membered ring. The question arises as to which of these crystal structures is energetically favoured. One might expect that the six-membered ring has a lower ring strain, while the dimers may allow denser packing of the molecules. The trimer has a molecular volume of 99.2 Å^3^ per formula unit (p.f.u.) H_2_B–N(CH_3_)_2_ under ambient conditions (Trefonas *et al.*, 1961 ▸). In the triclinic structure, the molecular volume of one formula unit of H_2_B–N(CH_3_)_2_ is 98.40 (5) Å^3^ at 100 K and increases to 107 (5) Å^3^ in the monoclinic structure at 295 K. In other words, the orthorhombic crystal structure of the six-membered rings allows a denser packing of the molecules under ambient conditions. If the energy difference between the two forms is calculated using dispersion-corrected DFT, both the PBE-D2 and the PBE-TS functionals favour the orthorhombic (trimer) structure, with energy differences of −6.4 kJ mol^−1^ p.f.u. and −0.6 kJ mol^−1^ p.f.u., respectively (Table 2 ▸). The rather large dependence on the dispersion correction scheme is noteworthy, especially as a separation of dispersion interactions from the DFT total energy results in a dispersion energy difference of +4.3 kJ mol^−1^ (favouring the dimer phase) for both functionals used. In other words, the inclusion of dispersion interactions brings the two phases closer together. Clearly, the rather large difference among the PBE-D2 and PBE-TS results indicates that higher-level methods would be needed to obtain a fully quantitative picture. However, it should be emphasized that both approaches predict the trimer to be a thermodynamically more stable form. This implies that the de­hydro­coupling of unit H_3_B–NH(CH_3_)_2_ kinetically favours the formation of the four-membered ring over the thermodynamically preferred six-membered ring.

## 4. Conclusion   

In this work, the phase transition from the dimeric triclinic di­methyl­amino­borane to a dimeric monoclinic structure was studied. The crystal structure of the monoclinic phase was determined from X-ray powder diffraction data. The unit-cell parameters and the space group *C*2/*m* fit very well to the work of Schapiro (1962 ▸). However, no fractional coordinates were given in that work. The phase transition from the dimeric triclinic to the dimeric monoclinic phase is of second order, accompanied by a spontaneous strain in the triclinic phase. From phonon calculations, an optical *B_g_* mode was calculated at a very low wavenumber of 8.06 cm^−1^. This mode can be identified to have a high contribution to the thermal expansion leading to the phase transformation. Upon cooling of the monoclinic phase, this mode freezes and reduces the symmetry from monoclinic (space group *C*2/*m*) to triclinic (space group symmetry 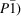. Di­methyl­amino­borane forms not just as a dimer but also as a trimer. Many group 13 element amino compounds form species consisting of either six-membered or four-membered rings, but di­methyl­amino­borane is one of the rare examples which can be found in both modifications. Comparing the energies obtained from DFT calculations in conjunction with a dispersion correction reveals that the six-membered ring is more stable than the four-membered ring. This can be understood in terms of ring strain in the four-membered ring. The reason why the four-membered ring is formed has to be a kinetic effect, since just two molecules are needed to form a four-membered ring, in contrast to the formation of a six-membered ring by three molecules. Another interesting result is that the energy difference between the four-membered and the six-membered rings becomes smaller if dispersion corrections are taken into account. This could mean that the packing of four-membered rings is more efficient than that of six-membered rings.

## Supplementary Material

Crystal structure: contains datablock(s) I. DOI: 10.1107/S2052520621001979/ra5089sup1.cif

Rietveld powder data: contains datablock(s) I. DOI: 10.1107/S2052520621001979/ra5089Isup2.rtv

CCDC reference: 2063825

## Figures and Tables

**Table 1:** Results of the Rietveld refinement of the monoclinic crystal structure of di­methyl­amino­borane [measured at 293 (2) K] with *TOPAS* together with the literature values of Schapiro (1962 ▸)

Experiment	This study	Schapiro (1962 ▸)
Crystal system, space group	Monoclinic, *C*2/*m*	Monoclinic, *C*2/*m*
*a* (Å)	6.2314 (3)	6.24
*b* (Å)	11.0574 (7)	11.07
*c* (Å)	6.2759 (4)	6.28
β (°)	98.754 (3)	98.8
*Z*	4	4
*V* (Å^3^)	427.39 (4)	428.7
No. of parameters	51	
No. of restraints	31	
2θ range (°)	3.00–59.67	
*R*_w_ (%)	5.5	
GoF	2.7	

**Table 2:** Comparison between the calculated and the experimental unit-cell parameters of dimeric and trimeric di­methyl­amino­borane, obtained in DFT calculations The errors of the extrapolated values are obtained from the fit of the unit-cell parameters against the temperature [cell choice is the same as that used by Jaska *et al.* (2001 ▸)].

		Experiment			
	0 K	At 100 K	At 298 K	PBE-D2	PBE-TS
Dimer					
*a* (Å)	5.72 (5)	5.8330 (7)	6.3460 (5)	5.740	5.695
*b* (Å)	5.93 (1)	6.029 (1)	6.236 (1)	5.688	5.844
*c* (Å)	6.207 (5)	6.240 (1)	6.2784 (3)	5.970	6.118
α (°)	81.0 (5)	80.372 (8)	81.25 (5)	81.41	81.03
β (°)	82.4 (8)	81.533 (1)	85.57 (5)	81.65	82.56
γ (°)	67.6 (1)	65.942 (8)	60.68 (2)	66.69	66.07
					
			At 298 K	PBE-D2	PBE-TS
Trimer					
*a* (Å)			11.2	10.737	11.037
*b* (Å)			13.17	12.987	13.073
*c* (Å)			8.07	7.672	7.731
Total energy difference p.f.u. (kJ mol^−1^)				−6.4	−0.6
Dispersion energy difference p.f.u. (kJ mol^−1^)				+4.3	+4.3
